# Genomic characterization of the emerging multidrug-resistant *Klebsiella pneumoniae* ST6668 clone in northern Italy, 2020–2025

**DOI:** 10.1128/spectrum.03499-25

**Published:** 2026-06-01

**Authors:** Maryam Omrani, Virginia Batignani, Kiarash Moghaddasi, Arash Ghodousi, Stefano Gaiarsa, Aurora Piazza, Francesca Saluzzo, Daniela Maria Cirillo

**Affiliations:** 1 Division of Immunology, Transplantation, and Infectious Diseases, IRCCS San Raffaele Scientific Institute 721820 , Milan, Italy; 2 Laboratory of Mycobacteriology, Department of Biology and Biotechnology “Lazzaro Spallanzani", University of Pavia 19001, Pavia, Italy; 3 Università Vita Salute San Raffaele 18985, Milan, Italy; 4 Microbiology and Virology Unit, Fondazione IRCCS Policlinico San Matteo 18631, Pavia, Italy; 5 S.C.C.D.P. Department, Microbiology and Clinical Microbiology Unit, University of Pavia 19001, Pavia, Italy; 6 I.R.C.C.S. Policlinico S. Matteo 18631, Pavia, Italy; Universita degli Studi dell'Insubria 19045, Varese, Italy

**Keywords:** *Klebsiella pneumoniae*, ST6668, NDM-1, ST147, hybrid assembly, multidrug resistance, Italy

## Abstract

Carbapenem-resistant *Klebsiella pneumoniae* carrying New Delhi metallo-β-lactamase (NDM) enzymes poses a major clinical and public health challenge due to their ability to spread rapidly in hospitals, and their resistance to most β-lactam antibiotics. Following the first report of the emerging NDM-producing *K. pneumoniae* lineage ST6668 in Pavia, Italy, we conducted a retrospective analysis at San Raffaele Hospital (Milan). We identified 87 ST6668 isolates, several of which were collected prior to the initial report from Pavia. Whole-genome sequencing data were used to compare the Milan isolates with the 52 genomes from Pavia, in order to characterize a total of 139 ST6668 isolates collected between 2020 and 2025 from three hospitals in northern Italy. Phylogenetic and comparative genomic analyses were performed based on single-nucleotide polymorphisms (core SNPs), including 310 ST147 genomes for comparison. Accessory genome profiling was further validated by hybrid assemblies of three representative strains. A phylogenetic cluster containing isolates from different hospitals suggested possible interhospital transmission. Although the origin of ST6668 remains unknown, it clustered closely with KL64-ST147. The hybrid assemblies revealed that *bla*_NDM-1_ is carried by the IncFIB(pQil) or IncHI1BIncFIB(Mar) plasmids. ST6668 lacks virulence markers such as *iuc* and *rmpADC*. All ST6668 isolates analyzed here carry a prophage identical to vB_Kpn_147Tu, which has been previously reported in ST147 isolates from the Tuscany outbreak. Together, these findings suggest either recent divergence from a common ancestor or convergence via mobile genetic elements.

**IMPORTANCE:**

Carbapenem-resistant *Klebsiella pneumoniae* producing New Delhi metallo-β-lactamase (NDM)-type enzymes represent a serious threat to infection control and patient safety. Understanding how novel resistant lineages emerge and spread is essential to limit hospital outbreaks. Our study traced the early presence of the recently identified ST6668 lineage in Milan, showing that it was circulating before its first description in Pavia. By linking this clone to the well-known high-risk lineage ST147, we demonstrate how resistance and genetic elements move between successful bacterial populations. These insights highlight how genomic surveillance can uncover hidden transmission pathways and guide interventions to prevent further dissemination of multidrug-resistant *K. pneumoniae* in healthcare settings.

## INTRODUCTION

Carbapenemase-producing Enterobacterales (CPE) are among the most alarming multidrug-resistant pathogens, with limited treatment options, high morbidity, and mortality rates (1, 2). *Klebsiella pneumoniae*, a leading cause of CPE infections, is designated a high-priority pathogen by the World Health Organization for its widespread resistance and clinical impact (3).

New β-lactam/β-lactamase inhibitor combinations, such as ceftazidime–avibactam, imipenem–relebactam, and meropenem–vaborbactam, have expanded treatment options for some CPE infections (4, 5). However, they are ineffective against metallo-β-lactamase (MBL) producing strains, especially those carrying *bla*_NDM_ genes associated with high risk and strong potential for horizontal gene transfer (HGT) (6–9).

In Italy, CPE has circulated endemically for over a decade, mainly driven by the clonal expansion of KPC-producing *K. pneumoniae*, particularly clonal complex 258 (CC258). More recently, other KPC-producing lineages, such as ST307 and ST101, have emerged (10–12). By contrast, MBL-producing *K. pneumoniae,* especially New Delhi metallo-β-lactamase (NDM) producers, were historically rare until the major 2018 Tuscany outbreak dominated by the ST147 lineage (13–16).

In 2023, a novel NDM-1-producing clone, ST6668, belonging to clonal complex 147 (CC147), was identified during a multi-hospital outbreak in Pavia, northern Italy. ST6668 showed broad resistance to β-lactams, aminoglycosides, and ceftazidime–avibactam. Genomic analyses placed ST6668 close to ST147, differing by a single-nucleotide polymorphism (SNP) in the *phoE* gene, raising important questions about its origin and regional spread (17).

Here, we investigate the emergence and phylogenetic context of ST6668 isolates identified at San Raffaele Hospital in Milan. Retrospective WGS of CPE *K. pneumoniae* isolates collected between January 2020 and June 2025 revealed ST6668 circulation prior to its identification in Pavia. We further characterize its population structure, compare it with ST147 lineages, and explore genomic features consistent with a shared evolutionary background and possible interregional transmission.

## MATERIALS AND METHODS

### Bacterial isolates and genomic data collection

At San Raffaele Hospital, all carbapenemase-producing *Klebsiella pneumoniae* (CPE) isolates recovered from clinical and screening samples are routinely collected and subjected to whole-genome sequencing. Between 2019 and 2025, we collected 527 unique *K. pneumoniae* CPE isolates. ST6668 isolates were identified using multilocus sequence typing based on whole-genome sequencing data and selected for this study. Only one isolate per patient was included, with the earliest available isolate retained in cases of multiple samples.

Overall, we analyzed 139 *Klebsiella pneumoniae* ST6668 genomes collected across three hospitals in northern Italy. Of these, 87 CPE isolates were collected at San Raffaele Hospital (Milan) between 2020 and 2025 and sequenced as part of this study. The remaining 52 genomes originated from the Pavia area (Voghera Hospital and Fondazione IRCCS Policlinico San Matteo) and were retrieved from publicly available whole-genome sequencing data sets collected in 2023 (17).

Among the 87 clinical isolates from San Raffaele, the most common samples were rectal swab (*n* = 39, 44.8%) and urine (*n* = 29, 33.3%). Other samples included blood (*n* = 8, 9.2%), bronchoalveolar lavage (*n* = 2, 2.3%), pus (*n* = 2, 2.3%), biopsy (*n* = 1, 1.1%), and bronchoaspirate (*n* = 2, 2.3%).

To contextualize ST6668, we analyzed 310 ST147 genomes: 48 from the 2018 Tuscany outbreak (PRJNA643814), 5 from Pisa (PRJNA667843, PRJNA746575) (18, 19), 33 from San Raffaele (2020–2024), and 223 selected via BV-BRC (bv-brc.org) using MASH (20) as described in the P-DOR pipeline (21). Duplicate genomes were removed.

### Illumina short-read DNA sequencing and analysis

Strains were grown on MacConkey or blood agar plates from the original clinical samples.

Genomic DNA was extracted using the Maxwell RSC Pathogen Total Nucleic Acid Kit (Promega, USA) and quantified with a Qubit fluorometer (Thermo Fisher Scientific, USA). Libraries were prepared with the Nextera XT v2 kit (Illumina, USA) and sequenced on an Illumina NextSeq 500 platform using the Mid Output Kit (300 cycles), generating 2 × 150 bp paired-end reads, as previously described (22–24).

### Nanopore long-read DNA sequencing and analysis

Three representative isolates were randomly selected for long-read sequencing to obtain complete plasmid sequences, as previously described (23). To recover large-sized DNA, genomic DNA was manually extracted. Extraction involved cell lysis using a custom-made lysis buffer supplemented with lysozyme (final concentration 10 mg/mL), followed by digestion with Proteinase K (final concentration 200 µg/mL), purification using phenol:chloroform:isoamyl alcohol (25:24:1, Tris-saturated, pH 8.0), and DNA precipitation with isopropanol and ethanol washes. In the next step, the Rapid Barcoding Sequencing kit 24 V14 (SQK-RBK114.24, Oxford Nanopore) was used according to the manufacturer’s protocol for genomic DNA. The sequencing was carried out on a portable MinION device using a flow cell R10.4. (Oxford Nanopore). Local basecalling was performed using Guppy (version 3.1.5) (Oxford Nanopore) with the option enabled to trim the sequencing adapters. NanoFilt (version 2.0.0) was used to filter out the reads with a Phred score < 7 and a length < 1,000 bp, and the statistics of the reads and quality scores were extracted with NanoStat (version 0.8.0) (25).

### Hybrid whole-genome assembly and quality control

Short-read-only assemblies were generated using Shovill v3.15.5 (github.com/tseemann/shovill). For isolates with both long- and short-read data, hybrid assemblies were performed using Canu v1.7.1 and Flye v2.9.1-b1780, producing two assemblies per isolate. The higher-quality assembly based on contiguity, circularization, and overall completeness was selected for each isolate. Assemblies were polished with multiple rounds of Racon v1.4.3 (long reads), followed by Pilon v1.23 (short reads). Contigs were filtered to retain sequences ≥300 bp with ≥5× coverage, and assembly quality was assessed using QUAST v5.2.0. Assemblies were visualized by Bandage v0.4.8 (26). Short reads were preprocessed with Trim Galore v0.6.5dev.

### *In silico* analysis and phylogeny

Assemblies were profiled with Kleborate v3.1.3 and PlasmidFinder v2.0.1 (DB 2023-01-18) to identify sequence types, resistance genes, and plasmid replicons.

Reads were mapped to complete genomes with BWA-mem2 v2.2.1 to validate accessory genome features identified by hybrid assemblies in isolates sequenced with short reads only.

Core genome SNPs were identified using Snippy v4.6.0 (27). A maximum likelihood phylogeny was inferred with RAxML v8.2.12 under the GTR+G model. Phylogenetic trees were visualized using iTOL (28).

## RESULTS

### Phylogenetic structure of ST6668

**Fig 1:**
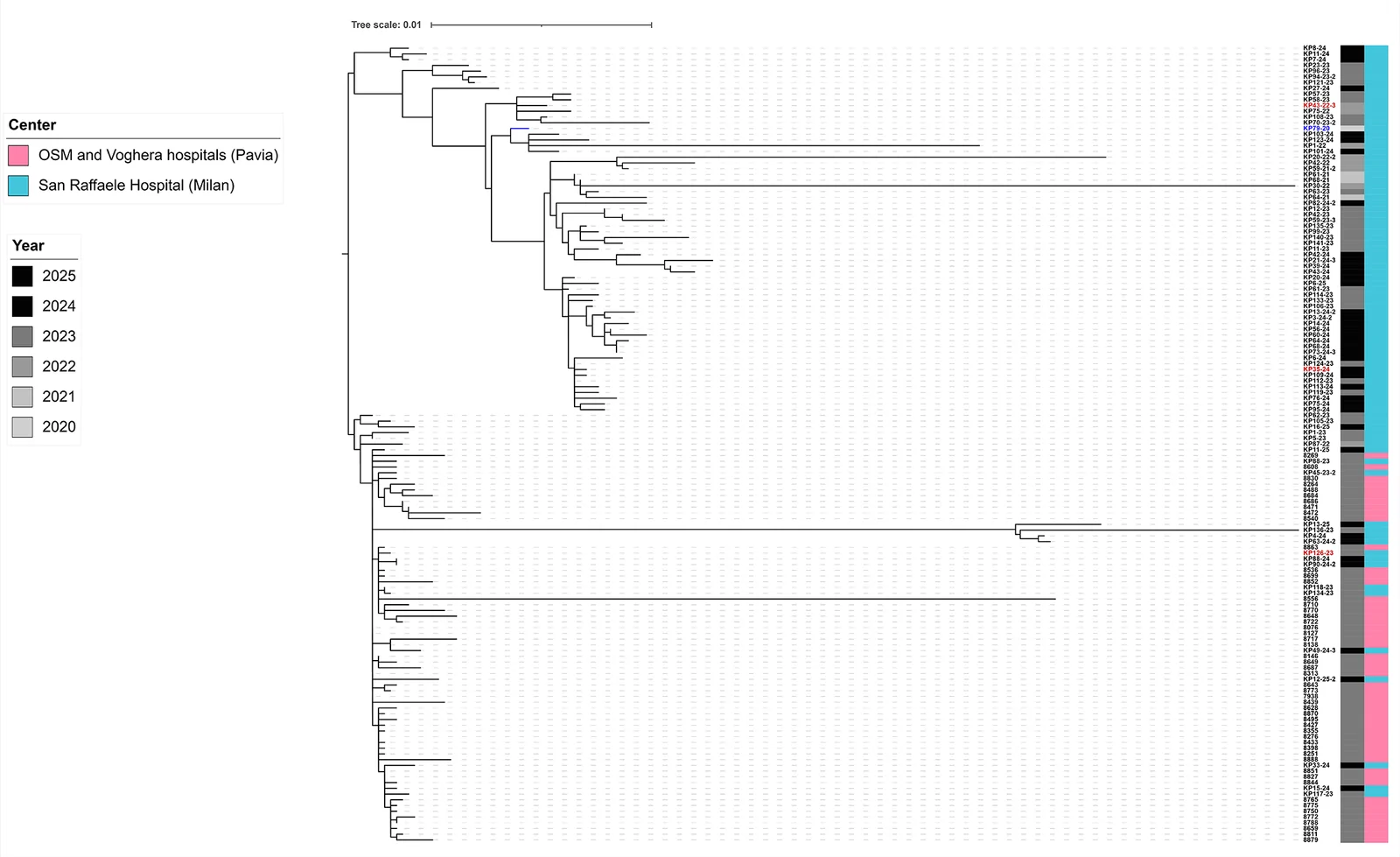
Core-genome maximum-likelihood phylogeny of ST6668 isolates from San Raffaele Hospital (Milan) and two hospitals in Pavia (Voghera and OSM). The tree was inferred from a core SNP alignment using a GTR+G substitution model and is midpoint-rooted for visualization purposes. The oldest ST6668 genome is highlighted in red. Three representative genomes subjected to hybrid assembly are highlighted in blue. Colored bars indicate the year of isolation and hospital of origin. The topology resolves two main clades: one composed exclusively of San Raffaele isolates, and a second containing isolates from San Raffaele together with isolates from both Pavia hospitals.

Core SNP-based phylogeny of ST6668 isolates from San Raffaele Hospital (Milan) and two hospitals in Pavia shows two distinct clades (Fig. 1). One clade is composed exclusively of isolates from San Raffaele Hospital, suggesting locally restricted diversification events that are likely unrelated to the Pavia outbreak. In contrast, the other clade includes isolates from both OSM and Voghera hospitals (Pavia), along with a subset of isolates from San Raffaele, suggesting a potential transmission link or a common source across these institutions.

The earliest ST6668 isolate in our data set (collected in 2020) is not basal in the phylogeny. Instead, both clades show high clonality, with isolates from different years. Longitudinal genomic surveillance will be required to determine whether this strain is a precursor to any later clades or simply represents a divergent but epidemiologically isolated lineage.

### Comparative phylogeny

To investigate the evolutionary relationship and potential origin of ST6668, we constructed a core genome phylogeny, including 449 isolates (310 ST147 + 139 ST6668). Although the tree does not point to a single origin, ST6668 forms a monophyletic clade closely related to KL64-ST147, largely from Italy (including isolates from the Tuscany outbreak). These ST147 isolates share the KL64 (wzi64) capsule locus, O2α O-antigen type, and predominantly carry the yersiniabactin lineage ybt9 on ICEKp3. However, a subgroup of ST147 genomes from various countries is located at the phylogenetic boundary with ST6668 and harbors ICEKp elements closely resembling those of ST6668.

**Fig 2:**
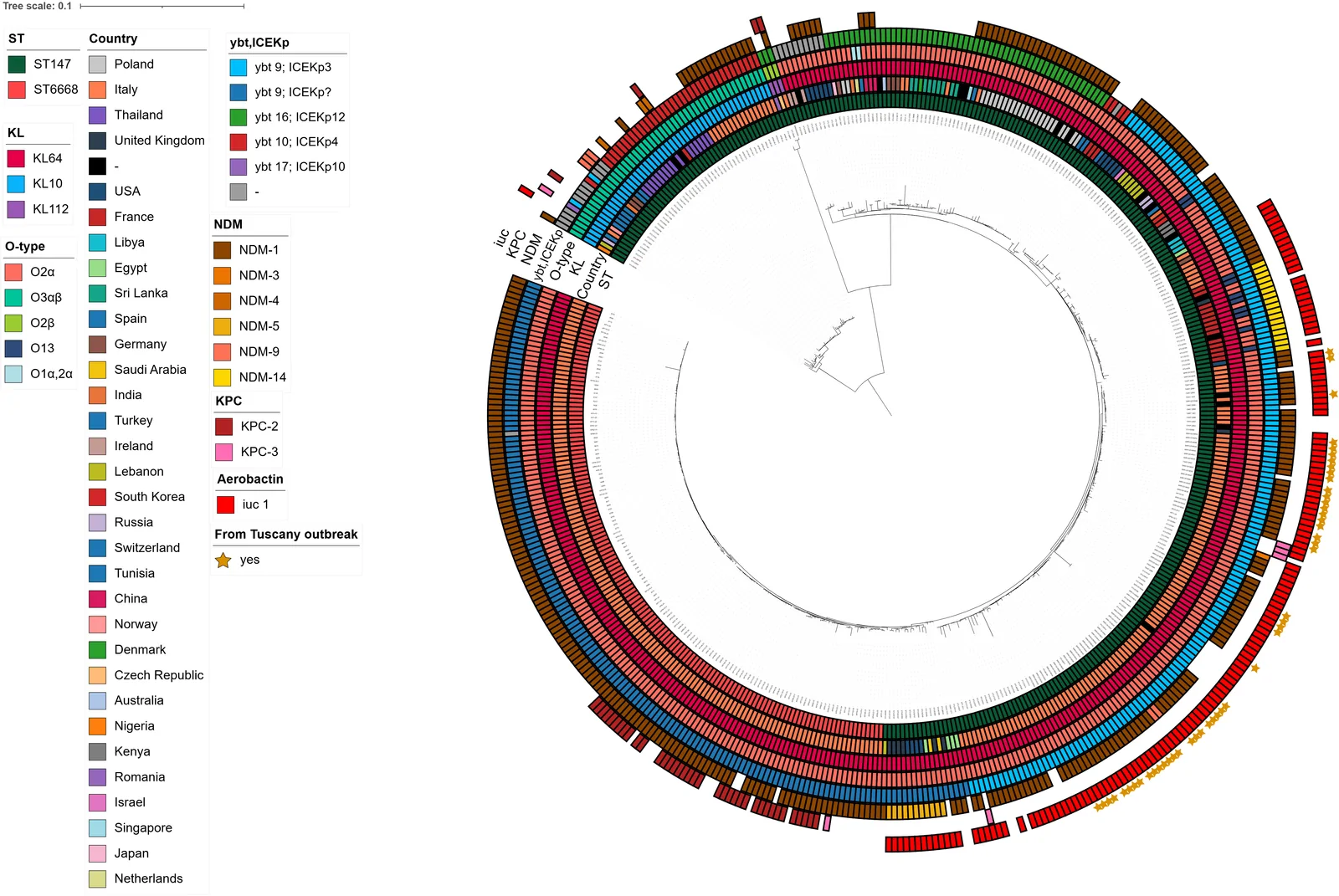
Core-genome maximum-likelihood phylogeny of ST6668 and background ST147 genomes. The tree was inferred from a core SNP alignment under a GTR+G model and is midpoint-rooted for visualization. ST6668 forms a distinct cluster closely related to KL64-ST147 circulating in Italy (including Tuscany strains), while a subset of non-Italian ST147, including Lebanese NDM-1 ST147, is positioned basally to ST6668. From inner to outer ring: sequence type (ST), country, capsule locus (KL), O-antigen type, yersiniabactin and ICEKp lineage, aerobactin (*iuc*) presence/absence, and Tuscany outbreak strains.

All ST6668 lacked virulence markers, such as the iuc aerobactin locus and the rmpADC operon (regulators of the mucoid phenotype). Furthermore, a distinct ST147 cluster, including strains from San Raffaele Hospital, belonged to KL10 (wzi420)–O3αβ with mostly ybt10 on ICEKp4, indicating the parallel evolution of two distinct ST147 groups within San Raffaele Hospital (Fig. 2). Kleborate summaries are in Table S1.

### Resistome profile

*In silico* resistome analysis revealed widespread carbapenemase gene carriage among ST6668 isolates from San Raffaele. The majority harbored *bla*_NDM-1_ either alone (*n* = 49, 56.3%) or in combination with *bla*_KPC-2_ (*n* = 34, 39.8%) or *bla*_KPC-3_ (*n* = 1, 1.1%). An additional 3.4% of the isolates carried *bla*_KPC-2_ alone. Extended-spectrum β-lactamase gene *bla*_CTX-M-15_ was detected in 45.9% (*n* = 40) of isolates.

The 16S rRNA methylase gene *armA,* conferring high-level aminoglycoside resistance, was found in (*n* = 43, 49%). Other acquired resistance genes included those associated with β-lactam resistance (*bla*_TEM-1_*, bla*_TEM-32_*, bla*_OXA-1_, and *bla*_OXA-9_), aminoglycosides (*aacA4, aadA1, aph[3*′*]-Ia,* and *aph[3*′*]-VI*), sulfonamides (*sul1* and *sul2*), trimethoprim (*dfrA5*), quinolones (*qnrS1*), rifampicin (*arr-3*), macrolides (*mph[A], mph[E], msr[E]*), and phenicols (*catB3*). The distribution of resistance genes across isolates is detailed in Table S1.

Phenotypic antimicrobial susceptibility data for ST6668 isolates are presented in Table S2.

### Accessory genomes

*In silico* plasmid replicon typing of draft genomes from ST6668 isolates collected at San Raffaele Hospital (Milan) revealed a diverse set of incompatibility groups, consistent with substantial genetic plasticity and HGT potential. The most prevalent replicons include Col440I, IncFIB(pKPHS1), IncHI1B(Mar), IncR, and IncFIB(Mar), mirroring the ST6668 plasmid profile from Pavia (17) (Table 1 and Table S3).

**TABLE 1:** Plasmid replicon prevalence in ST6668 *K. pneumoniae* from San Raffaele Hospital (Milan)

Replicon	Prevalence (%)
Col440I	100.0
IncFIB(pKPHS1)	95.4
IncHI1B(Mar)	85.1
IncR	80.5
IncFIB(Mar)	77.0
IncFIB(pQil)	64.4
IncFII(K)	44.8
IncFIB(K)	11.5
IncX1	6.9
IncFII	6.9
IncX3	4.6
Col(BS512)	2.3
ColRNAI	1.15
IncFIB(AP001918)	1.15
IncI1	1.15

Long-read sequencing of three ST6668 isolates (SanRaffaele126-23, SanRaffaele43-22, and SanRaffaele35-24) yielded complete circular assemblies resolving 11 accessory elements, including hybrid IncHI1B–FIB(Mar) and IncFII(K)–IncFIB(K) plasmids, single IncR and IncFIB(pQil) plasmids, and a phage genome (Table 2). Similar plasmid profiles were observed in previously reported NDM-1-producing ST147 from Tuscany (16) and Lebanon (29), which are clustered closely with ST6668.

**TABLE 2:** Summarizing the accessory genome elements retrieved through hybrid assemblies for representative ST6668 strains

Plasmid	Related strain	Size (bp)	Replicon	Resistance or virulence traits
R-126-6668-MI	126	39,711	R	*aacA4, bla*_OXA9*,*_ *bla*_TEM-1*,*_ *aadA1*, *aac(2′)-IIa*
pQil-NDM-126-6668-MI	126	54,064	FIB(PQil)	*aacA4-cr, bla*_NDM-1_ *, ble*_MBL_ *, bla*_CTX-M-15_ *, qnrS1, bla*_OXA1_ *, sul1, catB3*
HI1B-FIB-43-6668-MI	43	258,205	HI1B-FIB(Mar)	*bla*_NDM-1_*, bla*_TEM-1_*, armA, aacA4, sul1, sul2, dfrA5, dfrA22, dfrA10, bla*_OXA*9*_, *aadA1, msr(E), mph(E), emr(E), qnrS1, aph(3')-VI, ble*_MBL_, *bla*_OXA-948_, *bla*_OXA-895_, *bla*_IMP-37_, *bla*_SHV-22_, *aac(6′)-Ib10, tetA*, *tetB*, *bla*_CTX-45_, *bla*_OXA-445_, *bla*_OXA-129_
HI1B-FIB-35-6668-MI	35	244,051	HI1B-FIB(Mar)	*bla*_NDM-1_ *, armA, sul1, sul2, mph(A), msr(E), emr(E), aph(3')-VI, dfrA1*
R-35-6668-MI	35	48,817	R	*bla*_KPC-2_ *, bla*_OXA-9_ *, bla*_TEM-1_ *, aacA4, aadA1*
IncFII(K)-IncFIB(K)−35-6668-MI	35	230,602	IncFII(K)-IncFIB(K)	*sul2, aacA4, bla*_TEM-1_ *, aadA1, qnrB1, dfrA14, dfrA*
IncFII-43-6668-MI	43	15,829	IncFII	*tetA, armA, ompA*
IncX1-43-6668-MI	43	24,301	IncX1	*sul1, mph(A), emr(E), aadA5, dfrA17*
vB-6668-MI^a^		112,867		*dfrA*

^a^ Present in all three representative strains.

**Fig 3:**
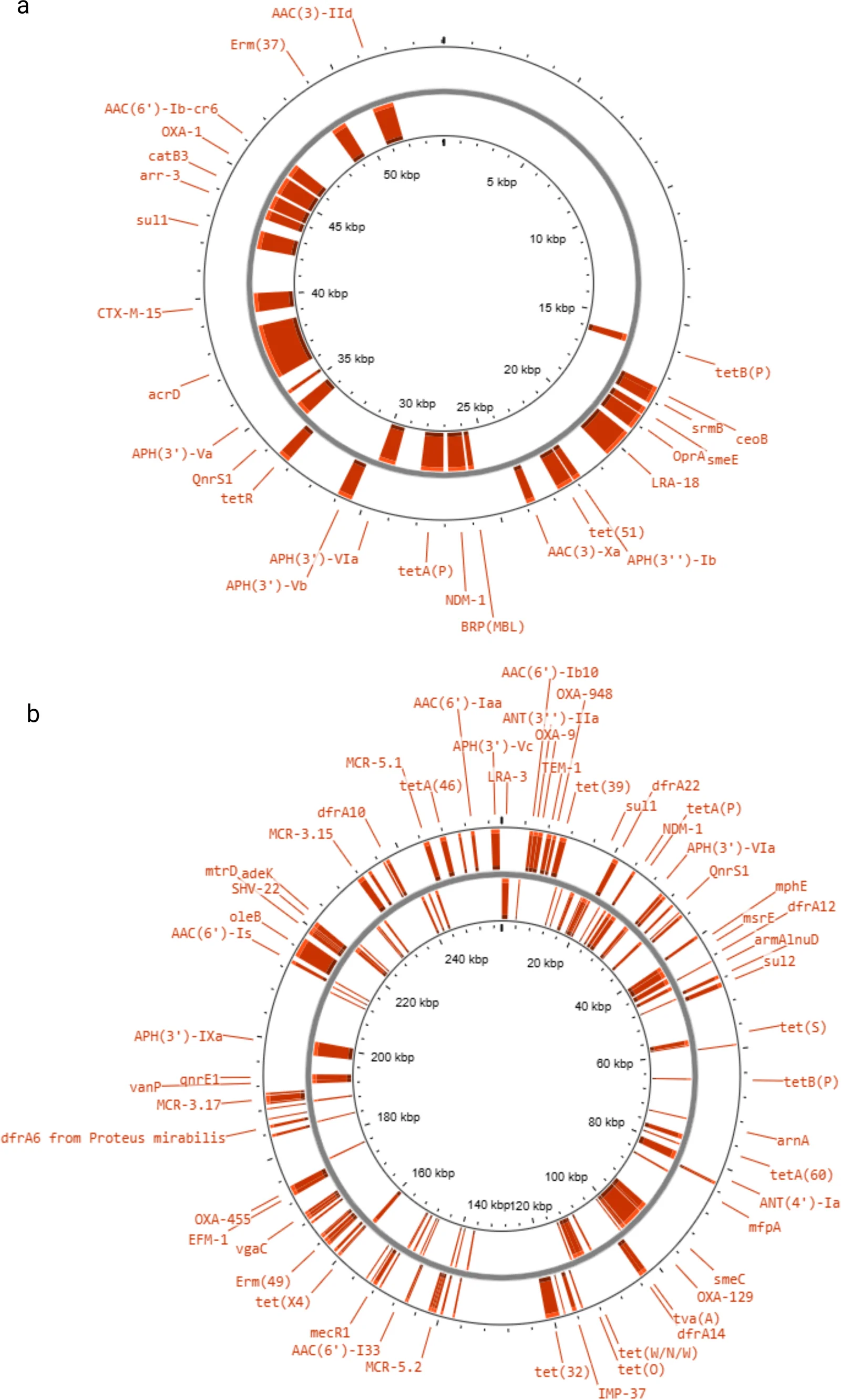
(**a**) Complete sequence of plasmid pQil-NDM-126-6668-MI retrieved by hybrid assembly and annotated for antimicrobial resistance genes. The circular map represents the fully assembled pQil-NDM-126-6668-MI plasmid obtained from a representative ST6668 isolate. Annotation was performed using the Comprehensive Antibiotic Resistance Database (CARD), highlighting the presence of key resistance genes, including *bla_NDM-1_*, *bla_CTX-M-15_*, *aac(6')-Ib-cr6*, *qnrS1*, *sul1*, and *catB3*. The blast results show 100% identity with CP071030 and >98% identity with part of NC_014016. (**b**) Circular representation of plasmid HI1B-FIB-43-6668-MI retrieved by hybrid assembly and annotated for antimicrobial resistance genes. This figure shows the complete sequence of the HI1B-FIB-43-6668-MI plasmid from a representative ST6668 isolate. Annotation using the CARD reveals a dense array of resistance determinants, including *bla_NDM-1_*, *armA*, *bla_TEM-1_*, *sul1*, *sul2*, *qnrS1*, *aadA1*, *aph(3')-VI*, *mph(E)*, *msr(E)*, and *dfrA5*.

One out of three isolates carried a *bla*_NDM-1_ IncFIB(pQil) plasmid (pQil-NDM-126-6668-MI) co-encoding *aac(6′)-Ib-cr* and *bla*_CTX-M-15_ (Fig. 3a), showing 99.99% identity to CP071030 (Tuscany ST147) and strong backbone similarity to the prototypical pKpQIL FII–FIB plasmid (NC_014016) (Fig. 3a), the latter known for global dissemination of KPC carbapenemases (30).

**Fig 4:**
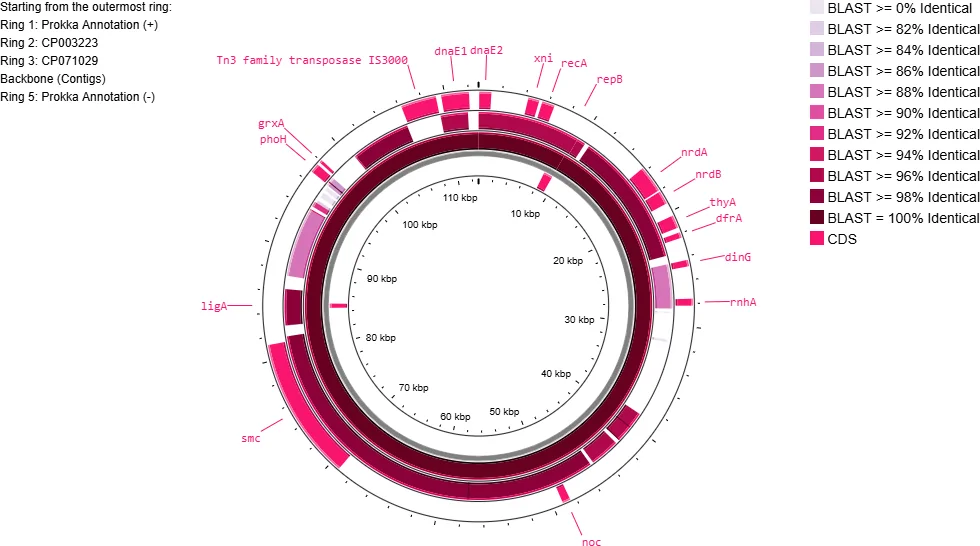
BLAST alignment of the accessory genome identified in representative ST6668 isolates with bacteriophage vB_Kpn_147Tu (GenBank accession CP071029) (identified during the Tuscany ST147 outbreak) and IncFIB(pKPHS1) plasmid (GenBank accession CP003223). The blast results show 100% identity and identical length with CP071029, supporting the presence of a shared prophage element. This region also overlaps with IncFIB(pKPHS1) plasmid sequences CP003223, suggesting a phage-plasmid mosaic structure.

Two other representative isolates carried *bla*_NDM-1_ on IncHI1B–FIB(Mar) with *armA*, *sul1,* and *sul2* (Fig. 3b). However, virulence genes that have been reported on the same plasmid from Tuscany (16) and Lebanon were not detected (29).

An IncR plasmid found in two out of three isolates carried *bla*_KPC-2_, *bla*_OXA-9_, and *bla*_TEM-1_ but lacked *bla*_NDM-1_, unlike IncR plasmids in Tuscany ST147 isolates.

A conserved phage genome identical to CP071029 (17) was present across all ST6668 (Fig. 4). Accessory-element presence in remaining isolates was confirmed by short-read mapping (≥95% length coverage; Table S3).

## DISCUSSION

The emergence of *Klebsiella pneumoniae* ST6668 represents a significant development in the epidemiology of CPE in Italy. By integrating additional ST6668 genomes from Milan with publicly available data from Pavia, this study expands the initial description of ST6668 and places it within a broader genomic and epidemiological context. Comparative analysis with the high-risk lineage ST147, together with in-depth characterization of the accessory genome, provides insights into the evolutionary origins, dissemination dynamics, and resistance potential of this emerging clone.

Our data demonstrate that ST6668 was already circulating in Milan in 2020, predating its recognition during the 2023 Pavia outbreak. This finding indicates that ST6668 had likely been silently circulating at low prevalence prior to epidemic expansion. However, the earliest Milan isolate is not basal in the phylogeny, suggesting that it did not represent the founding genotype of the broader ST6668 population analyzed in this study. This observation argues against a single-point origin within one institution and instead supports multiple introductions or unsampled diversity preceding detection.

Core SNP analysis revealed a large phylogenetic cluster that includes isolates from all three hospitals, consistent with regional dissemination across northern Italy. While the presence of closely related isolates in different institutions raises the possibility of inter-facility transmission, the lack of a robust temporal signal precludes confident inference of transmission directionality. To this end, ongoing molecular clock reconstruction incorporating additional ST6668 genomes from other Italian centers may help clarify whether spread occurred through patient transfer, healthcare-associated networks, or repeated external introductions.

Phylogenetically, ST6668 forms a clade closely related to Italian ST147, one of the most successful and globally disseminated high-risk *K. pneumoniae* lineages. However, the presence of non-Italian ST147 genomes (sharing the same ICEKp lineage) between ST147 and ST6668 argues against simple local divergence. Instead, these data support a scenario of international introduction, followed by local establishment and expansion. This hypothesis is further supported by the similarity in plasmid content between ST6668 and NDM-1-producing ST147 isolates from Tuscany and Lebanon (16, 29).

Most ST6668 isolates from San Raffaele hospital carried *bla*_NDM-*1*_ either alone or in combination with *bla*_KPC-2_, whereas a smaller subset harbored *bla*_KPC-2_ alone. Co-production of NDM-1 and KPC-2 was observed in ST6668 isolates from other hospitals in Milan (31), but not among ST6668 from Pavia, whereas NDM-1 and KPC-3 were rarely detected. Notably, this distribution differs from the broader epidemiology in Italy, in which KPC-3 is typically more prevalent than KPC-2 (32), suggesting lineage-specific acquisition of carbapenemase determinants in ST6668.

Hybrid short- and long-read sequencing enabled complete genomes and resolution of complex plasmids (e.g., HI1B–FIB[Mar], IncFII[K]–IncFIB[K]), which are often fragmented by short reads. To our knowledge, this represents the first application of hybrid genome assembly to *K. pneumoniae* ST6668, providing high-resolution insight into its mobile genetic elements. The hybrid assembly revealed that *bla*_NDM-1_ was carried on different plasmids within our ST6668 isolates. In SanRaffaele126-23, it was located on FIB(PQil) plasmids, highly similar to the FIB(PQil) plasmid that was described in the ST147 Tuscany outbreak. In SanRaffaele35-24 and SanRaffaele43-22, NDM-1 was encoded on IncFIB(Mar) and IncHI1B_1_pNDM-MAR replicon plasmids. This family is characterized by high plasticity, with frequent and wide recombination events. In SanRaffaele35_24, KPC2 was carried on IncR, a combination rarely seen in Italy. We also detected a prophage identical to vB_Kpn_147Tu, previously seen in Tuscany ST147, sharing regions with IncFIB(pKPHS1), CP003223, suggesting possible prophage-plasmid mosaicism. Without hybrid assembly, this element would likely have been misclassified as an IncFIB(pKPHS1) plasmid, highlighting the risk of misinterpretation when relying on short-read data alone. This underscores the value of hybrid sequencing for mobile-element characterization.

ST6668 lacks aerobactin (iuc) and rmp (RMP) loci found in Tuscany and Lebanese ST147, implying the absence of a typical hypervirulence plasmid as previously observed in the Pavia isolates.

The detection of ST6668 across multiple hospitals, including evidence of earlier circulation, suggests interhospital transmission and highlights the need for coordinated genomic surveillance. Its silent spread since at least 2020 indicates that emerging high-risk clones may remain undetected. The coexistence of multiple carbapenemase genes and diverse plasmids further raises concerns about horizontal gene transfer and ongoing dissemination, underscoring the need for strengthened infection control measures.

This study is limited by its local scope and restricted time frame, which may constrain generalizability and transmission inference. Nevertheless, these findings underscore the need for multicenter genomic surveillance integrated with robust infection prevention measures to track dissemination and inform control strategies beyond the local context.

In conclusion, the regional spread of blaNDM-1-producing ST6668 across three northern Italian centers is concerning, given its broad resistance and limited therapeutic options.

## Supplementary Material

Reviewer comments

## Data Availability

Short-read sequencing data for 87 ST6668 *Klebsiella pneumoniae* isolates are available under BioProject accession PRJNA1345544. In addition, long-read sequencing data for three representative isolates are also available within the same BioProject.

## SUPPLEMENTAL MATERIAL

Table S1 spectrum.03499-25-s0001.xlsx Kleborate results for 449 genomes (310 ST147 + 139 ST6668).

Table S2 spectrum.03499-25-s0002.xlsx Phenotypic antimicrobial susceptibility data for ST6668 isolates.

Table S3 spectrum.03499-25-s0003.xlsx *In silico* plasmid replicon typing of draft genomes from ST6668 isolates.

OPEN PEER REVIEW reviewer-comments.pdf An accounting of the reviewer comments and feedback.

ASM does not own the copyrights to Supplemental Material that may be linked to, or accessed through, an article. The authors have granted ASM a non-exclusive, world-wide license to publish the Supplemental Material files. Please contact the corresponding author directly for reuse.
