# Supplementary material for: Genomic characterization of the emerging multidrug-resistant Klebsiella pneumoniae ST6668 clone in northern Italy, 2020–2025
Source: Microbiol Spectr. 2026 Jun 1;14(7):e03499-25. doi: 10.1128/spectrum.03499-25 (PMC13340089; doi:10.1128/spectrum.03499-25)
Supplement: Reviewer comments [file reviewer-comments.pdf]

AMERICAN SOCIETY FOR MICROBIOLOGY | Microbiology Spectrum

Genomic characterization of the emerging multidrug-resistant *Klebsiella pneumoniae* ST6668 clone in northern Italy, 2020–2025

Maryam omrani, Virginia Batignani, Kiarash Moghaddasi, Arash Ghodousi, Stefano Gaiarsa, Aurora Piazza, Francesca Saluzzo, and Daniela Maria Cirillo

Corresponding Author(s): Virginia Batignani, IRCCS San Raffaele Hospital

Review Timeline:

Submission Date:	November 3, 2025
Editorial Decision:	January 27, 2026
Revision Received:	March 23, 2026
Accepted:	March 26, 2026

Editor: Gabriele Arcari

Reviewer(s): The reviewers have opted to remain anonymous.

Transaction Report:

(Note: With the exception of the correction of typographical or spelling errors that could be a source of ambiguity, letters and reports are not edited. The original formatting of letters and referee reports may not be reflected in this compilation.)

DOI: https://doi.org/10.1128/spectrum.03499-25

Re: Spectrum03499-25 (Genomic characterization of the emerging multidrug-resistant *Klebsiella pneumoniae* ST6668 clone in northern Italy, 2020-2025)

Dear Dr. Daniela Maria Cirillo:

I would like to apologize for the delay in the reviewing process and thank you for the privilege of reviewing your work. Below you will find my comments, instructions from the Spectrum editorial office, and the reviewer comments.

Please return the manuscript within 60 days; if you cannot complete the modification within this time period, please contact me. If you do not wish to modify the manuscript and prefer to submit it to another journal, notify me immediately so that the manuscript may be formally withdrawn from consideration by Spectrum.

Revision Guidelines
To submit your modified manuscript, log into the submission site at https://spectrum.msubmit.net/cgi-bin/main.plex. Go to Author Tasks and click the appropriate manuscript title to begin. The information you entered when you first submitted the paper will be displayed; update this as necessary. Note the following requirements:

• Upload point-by-point responses to the issues raised by the reviewers in a file named "Response to Reviewers," NOT in your cover letter.
• Upload a compare copy of the manuscript (without figures) as a "Marked-Up Manuscript" file.
• Upload a clean .DOC/.DOCX version of the revised manuscript and remove the previous version.
• Each figure must be uploaded as a separate, editable, high-resolution file (TIFF or EPS preferred), and any multipanel figures must be assembled into one file.
• Any supplemental material intended for posting by ASM should be uploaded with their legends separate from the main manuscript. You can combine all supplemental material into one file (preferred) or split it into a maximum of 10 files with all associated legends included.

For complete guidelines on revision requirements, see our Submission and Review Process webpage. Submission of a paper that does not conform to guidelines may delay acceptance of your manuscript.

Data availability: ASM policy requires that data be available to the public upon online posting of the article, so please verify all links to sequence records, if present, and make sure that each number retrieves the full record of the data. If a new accession number is not linked or a link is broken, provide Spectrum production staff with the correct URL for the record. If the accession numbers for new data are not publicly accessible before the expected online posting of the article, publication may be delayed; please contact production staff (Spectrum@asmusa.org) immediately with the expected release date.

Publication Fees: For information on publication fees and which article types are subject to charges, visit our website. If your manuscript is accepted for publication and any fees apply, you will be contacted separately about payment during the production process; please follow the instructions in that e-mail. Arrangements for payment must be made before your article is published.

ASM Membership: Corresponding authors may join or renew ASM membership to obtain discounts on publication fees. Need to upgrade your membership level? Please contact Customer Service at Service@asmusa.org.

The ASM Journals program strives for constant improvement in our submission and publication process. Please tell us how we can improve your experience by taking this quick Author Survey.

Thank you for submitting your paper to Spectrum.

Sincerely,
Gabriele Arcari
Editor
Microbiology Spectrum

Reviewer #1 (Comments for the Author):

Dear Authors,
References cited in the Introduction should be carefully reviewed.
Spacing and formatting issues:
There are widespread spacing errors and merged words throughout the manuscript. All spacing between words and numbers

must be corrected systematically.

Bacterial names:
All bacterial names should be italicized throughout the manuscript. The full species name should be provided at first mention (e.g., Klebsiella pneumoniae), and subsequently, the abbreviated form (e.g., K. pneumoniae) should be used consistently.

Gene nomenclature:
All β-lactamase genes must follow standard formatting. For example, bla should be italicized, while the specific allele (e.g., NDM) should be non-italic and subscript. This format must be applied consistently across the text, tables, and figures.

Typographical errors in Table 2:
Several gene names in Table 2 are misspelled. The authors should carefully check all gene names and correct them according to the annotation outputs from bioinformatic tools.

Reference issues:
The reference list and in-text citations are inconsistent. References 11 and 12, in particular, are not cited anywhere in the text. Overall, reference formatting should be revised to follow the journal's style.

Figures quality:
Figures-especially Figures 1 and 2-are of low quality and difficult to read. High-resolution images must be provided.

Discussion section:
The Discussion is very short and superficial. It should be expanded to provide deeper interpretation, comparison with previous studies, and relevant references. Epidemiological implications should be discussed more thoroughly.
The discussion section is not well-referenced.

Limitations of the study:
The manuscript does not discuss the limitations of the study. A Limitations section should be added.

Reviewer #4 (Comments for the Author):

The authors performed whole-genome sequencing analysis of 87 NDM-1-producing K. pneumoniae strains belonging to ST6668 detected at a single institution in Milan, Italy, from 2020 to 2025. They integrated and analyzed this data with previously reported whole-genome sequencing data of NDM-producing K. pneumoniae ST6668 strains from two institutions in Pavia, Italy. These strains showed high similarity to the NDM-1-producing KL64-ST147 strain that caused an outbreak in Tuscany, but they lacked virulence genes such as rmpADC or iuc, which were present on the IncHIB-FIB(Mar) plasmid in those strains.
This study provides important information on the molecular epidemiology of multidrug-resistant K. pneumoniae. Below are suggestions for improving the description of methods and presentation of results.

Minor comments:
- Lines 75-87: The reviewer infer that the authors collected the 87 San Raffaele strains and performed whole-genome sequencing analysis while the registered whole-genome sequencing data was used for the 52 Pavia strains. If my inference is correct, the authors should clearly distinguish the strains they collected versus the strains for which they just utilized data in the text to avoid confusion.
- Lines 75-87: The method for collecting the 87 San Raffaele strains should be briefly explained. I would like to know the total number of carbapenem-resistant K. pneumoniae isolates detected during the study period, the method for selecting the ST6668 strain. Additionally, were the analyzed strains limited to one per patient? Such information would be useful for determining the epidemiological significance of the ST6668 strain analyzed in this study.
- Lines 97-99: It is stated that strains for long-read sequencing were selected randomly, but it is inferred that they were selected based on some criteria derived from short-read sequencing data (if truly selected completely randomly, this would be inappropriate). The method used for selecting the target strains should be described. Additionally, marking the selected strains in Figure 1 or Figure 2 would aid reader comprehension.
- Figure 2: Adding information about carbapenemase genes would be beneficial for discussing the clonal spread and horizontal transmission of resistance genes.
- Table 2: Information should be added indicating in which of the three strains each plasmid was present.
- Figures 3 and 4: Creating structural comparison diagrams with similar plasmids mentioned in the text would increase the information provided.
- Figure 4: blaIMP-37, blaOXA-129, blaOXA-455, blaSHV-22, etc., which are not listed in Table 2, are shown. Please verify the accuracy of the information.

Reviewer 1

References in the Introduction
We thank the reviewer for this observation. The references cited in the Introduction have been carefully reviewed. In particular, References 11 and 12 were verified and are correctly assigned to the corresponding text (line 122).

Spacing and Formatting Issues
We appreciate the reviewer's comment. All spacing errors and merged words throughout the manuscript have been systematically identified and corrected.

Bacterial Names
We thank the reviewer for highlighting this point. All bacterial names have been italicized throughout the manuscript. The full species name (e.g., *Klebsiella pneumoniae*) is provided at first mention, and the abbreviated form (e.g., *K.* pneumoniae) is used consistently thereafter.

Gene Nomenclature
We appreciate this suggestion. The formatting of β-lactamase genes has been standardized across the manuscript in accordance with accepted nomenclature conventions.

Typographical Errors in Table 2
We thank the reviewer for pointing this out. All gene names in Table 2 have been carefully reviewed and corrected.

Figures Quality
We appreciate the reviewer's comment. Figures 1 and 2 have been re-generated at higher resolution to improve readability.

Discussion Section
We thank the reviewer for this valuable comment. The Discussion section has been substantially expanded. Additional interpretation, comparison with previous studies (lines 323–327, 328–374, 378–383), and relevant references (Refs. 30–31) have been incorporated. The epidemiological implications of the findings have also been further elaborated (lines 391–395).

Limitations of the Study
We appreciate this suggestion. A dedicated Limitations section has been added to the manuscript (lines 455–458).

Reviewer 2

Lines 75–87
We thank the reviewer for this insightful comment.
The reviewer correctly inferred that 87 isolates were collected and sequenced at San Raffaele Hospital, while

52 genomes from Pavia were obtained from publicly available datasets. This distinction has now been clearly stated in the Methods section (lines 151–162).

We appreciate the reviewer's request for clarification. Additional details have been included in the Methods section regarding:
• the total number of carbapenem-resistant *K. pneumoniae* isolates identified during the study period,
• the criteria used for selecting ST6668 isolates, and
• the inclusion of only one isolate per patient.
These additions clarify the epidemiological context of the analyzed strains.

Lines 97–99 (selection of strains for long-read sequencing)
We thank the reviewer for raising this point. Three representative isolates were selected randomly to capture plasmid diversity. Hybrid assemblies were generated using two approaches, and for each isolate, the best-quality assembly was retained (lines 200–201). The selected isolates are now indicated in Figure 1.

Figure 2
We appreciate this suggestion. Information on carbapenemase genes has been added to Figure 2 to facilitate discussion of clonal spread and horizontal gene transfer.

Table 2
We thank the reviewer for this comment. Information specifying in which of the three representative strains each plasmid is present has been added to Table 2 (column: "Related strain").

Figures 3.a
We appreciate the reviewer's suggestion. Structural comparison diagrams with similar plasmids referenced in the text have been added to enhance the informational value of these figures. Figure 4 already contained part of this information and has been refined for clarity.

Figure 3.b (gene discrepancies)
We thank the reviewer for highlighting this issue. The presence of genes such as *blaIMP-37*, *blaOXA-129*, *blaOXA-455*, and *blaSHV-22* has been carefully reviewed. Inconsistencies between Figure 4 and Table 2 have been corrected, and Table 2 has been updated accordingly.

Re: Spectrum03499-25R1 (Genomic characterization of the emerging multidrug-resistant *Klebsiella pneumoniae* ST6668 clone in northern Italy, 2020-2025)

Dear Dr. Virginia Batignani:

Your manuscript has been accepted, and I am forwarding it to the ASM production staff for publication. Your paper will first be checked to make sure all elements meet the technical requirements. ASM staff will contact you if anything needs to be revised before copyediting and production can begin. Otherwise, you will be notified when your proofs are ready to be viewed.

Data Availability: ASM policy requires that data be available to the public upon online posting of the article, so please verify all links to sequence records, if present, and make sure that each number retrieves the full record of the data. If a new accession number is not linked or a link is broken, provide production staff with the correct URL for the record. If the accession numbers for new data are not publicly accessible before the expected online posting of the article, publication may be delayed; please contact ASM production staff immediately with the expected release date.

Publication Fees: For information on publication fees and which article types have charges, please visit our website. We have partnered with Copyright Clearance Center (CCC) to collect author charges. If fees apply to your paper, you will receive a message from no-reply@copyright.com with further instructions. For questions related to paying charges through RightsLink, please contact CCC at ASM_Support@copyright.com or toll free at +1-877-622-5543. CCC makes every attempt to respond to all emails within 24 hours.

ASM Membership: Corresponding authors may join or renew ASM membership to obtain discounts on publication fees. Need to upgrade your membership level? Please contact Customer Service at Service@asmusa.org.

Embargo Policy: A press release may be issued as soon as the manuscript is posted on the Spectrum Latest Articles webpage. The corresponding author will receive an email with the subject line "ASM Journals Author Services Notification" when the article is available online.

The ASM Journals program strives for constant improvement in our submission and publication process. Please tell us how we can improve your experience by taking this quick Author Survey.

Thank you for submitting your paper to Spectrum.

Sincerely,
Gabriele Arcari
Editor
Microbiology Spectrum
